# Metabolomic Profile Differences Between Demented and Non-Demented APOE4 Carriers in the Long Life Family Study

**DOI:** 10.1093/geroni/igab046.2224

**Published:** 2021-12-17

**Authors:** Mohit Jain, Joanne Murabito, Joseph Zmuda, Kaare Christensen, Michael Province, Zhezhen Jin, Joseph Lee, Yujing Yao

**Affiliations:** 1 University of California at San Diego, La Jolla, California, United States; 2 Boston University, Framingham, Massachusetts, United States; 3 University of Pittsburgh, Pittsburgh, Pennsylvania, United States; 4 Department of Public Health, University of Southern Denmark, Odense, Syddanmark, Denmark; 5 Washington University School of Medicine, Washington University School of Medicine, Missouri, United States; 6 Columbia University, New York, New York, United States; 7 Columbia University Mailman School of Public Health, New York, New York, United States

## Abstract

The apolipoprotein ε4 (APOE4) is the most prevalent genetic risk factor for late-onset Alzheimer’s Disease (AD). Here we assessed the metabolomic profile differences between APOE4 carriers who develop AD vs. who do not in a sample of 142 participants, aged 65-99 years in the Long Life Family Study (LLFS). Of 7,321 metabolites, we applied a generalized estimating equation model and identified 137 metabolites significantly associated with AD. Subsequent multivariate analyses were performed for prediction and clustering recognition. Among annotated metabolites, 8 metabolites in the eicosanoids and docosanoids group, 3 metabolites in the fatty acids group, and arabitol were associated with elevated risks of AD (OR: 1.6-2.3). On the other hand, a different set of metabolites were associated with reduced risks of AD (OR: 0.34-0.64). These metabolomic profile differences can be used to help with early diagnosis in the population of older APOE4 carriers in the pre-clinical stage.

